# The In Vivo Effect of Amorphous Drug Nanoprecipitates on the Intestinal Absorption of the PROTACs ARV-110 (Bavdeglutamide) and ARV-471 (Vepdegestrant)

**DOI:** 10.1021/acs.molpharmaceut.6c00207

**Published:** 2026-05-01

**Authors:** Janis Niessen, Mirko Koziolek, Anura Indulkar, Thomas Borchardt, Markus Sjöblom, Mikael Hedeland, Hans Lennernäs, David Dahlgren

**Affiliations:** † Department of Pharmaceutical Biosciences, Translational Drug Discovery and Development, 8097 Uppsala University, Uppsala 751 05, Sweden; ‡ Synthetic Molecules CMC R&D, AbbVie Deutschland GmbH & Co. KG, Knollstrasse, Ludwigshafen am Rhein 67061, Germany; § AbbVie Small Molecule CMC Development, 1N Waukegan Road, North Chicago 07065, Illinois, United States; ∥ AbbVie Product Development Science and Technology, 1N Waukegan Road, North Chicago 07065, Illinois, United States; ⊥ Department of Medical Cell Biology, 8097 Uppsala University, Uppsala 751 05, Sweden; # Department of Medicinal Chemistry, Analytical Pharmaceutical Chemistry, Uppsala University, Uppsala 751 05, Sweden

**Keywords:** PROTAC, nanoparticle, particle drifting effect, pharmacokinetics, bioavailability, absorption

## Abstract

Targeted degradation of disease-associated proteins via proteolysis-targeting chimeras (PROTACs) is a powerful pharmacological strategy, but it requires a complex molecular architecture associated with poor intestinal absorption. Rational development of orally bioavailable PROTACs requires a thorough understanding of their biopharmaceutical properties. This mechanistic in vivo study aimed to address the potential of colloidal particle formulations to enhance the intestinal absorption of two model PROTACs, ARV-110 and ARV-471. Dose escalation studies were conducted in rats to investigate the impact of particle size and concentration on intestinal absorption and bioavailability of the PROTACs. The contribution of colloidal amorphous drug nanoprecipitates was moreover directly quantified by comparing intestinal absorption from a colloid-forming formulation with that of an amorphous, noncolloidal counterpart. All in vivo experiments were complemented by in vitro determinations of amorphous solubility, solubilization dynamics, and characterization of the size and solid state of the emerging colloidal particles. The two PROTACs consistently formed luminally stable, amorphous drug nanoprecipitates in vivo. Dose-proportional increases in absorption were observed at concentrations up to 15-fold above their amorphous solubility. The amorphous drug nanoprecipitates directly contributed to an enhanced absorptive flux up to 7-fold compared to a noncolloidal amorphous powder. The potentiating effect was attributed to particle drifting of the nanoparticles across the aqueous boundary layer, which increased the free drug concentration at the epithelial membrane. For the less soluble ARV-110, the colloid effect was capped at doses >0.2 mg/kg due to saturation. For the more soluble ARV-471, the colloid effect persisted at doses up to 5.0 mg/kg but at a reduced rate due to the formation of larger, less mobile particles. Overall, this work provided mechanistic insight into PROTAC absorption and suggested that formulations capable of generating stable amorphous drug nanoprecipitates represent a promising strategy to enhance the oral bioavailability of low-solubility PROTACs.

## Graphical abstract

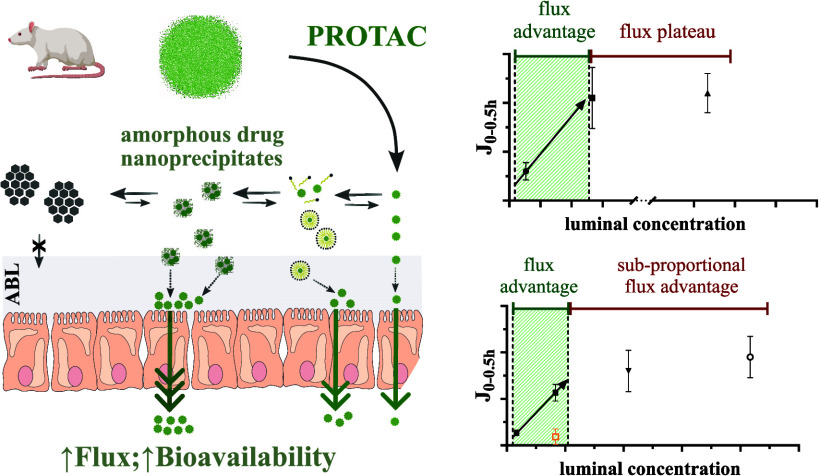

## 1 Introduction

As biological targets grow increasingly complex, drug discovery efforts are progressively generating structurally intricate molecules that often fall outside the traditional drug-like chemical space originally defined by Lipinski’s Rule of Five.^1,2^ Among these emerging drug modalities, proteolysis-targeting chimeras (PROTACs) have garnered significant attention. PROTACs represent a novel therapeutic strategy that harnesses the intracellular ubiquitin–proteasome system to selectively degrade disease-relevant proteins by forming a ternary complex with the target protein and an E3 ubiquitin ligase.^3–7^ Complexation requires three molecular elements: (i) a ligand for the target protein, (ii) a ligand for the E3 ligase, and (iii) a linker connecting the two. This architecture typically results in large (>700 Da), lipophilic molecules (log*p* > 4) with high polar surface areas (PSA > 180 Å^2^) and significant conformational flexibility (rotatable bonds >7).^8^ These properties tend to translate into poor biopharmaceutical properties, in accordance with class II or IV of the biopharmaceutics classification system (BCS).^9–11^

In vitro assays consistently report low to undetectable permeation of PROTACs,^12–14^ and very low aqueous solubility (<3 μM).^11,12^ This further renders the application of in vitro models such as PAMPA and cell-based assays unpredictive due to recovery and solubility limitations.^15,16^ The predominantly poor solubility and permeability are reflected in vivo, where a majority of 1806 PROTACs evaluated in rats exhibit intestinal absorption below 20%.^17^ Nonetheless, we have reported in vivo effective intestinal permeability values of PROTACs in rats consistent with human fraction absorbed above 0.35 and 0.85 for ARV-471 and ARV-110, respectively,^18^ despite their negligible in vitro flux.^13,19,20^ The lipophilic, flexible nature of PROTACs confers a high solubilization propensity in intestinal fluids, along with a high supersaturation potential and a low tendency for crystallization.^20–23^ These attributes highlight opportunities for enabling oral formulations and drug delivery strategies to enhance the oral bioavailability of low-solubility PROTACs.

As illustrated in Figure 1, upon dissolution, PROTACs can supersaturate intestinal fluids up to their amorphous solubility, representing the maximum achievable solute activity and the primary thermodynamic driving force for passive intestinal membrane permeation. Exceeding the amorphous solubility may result in precipitation to a crystalline solid state that is not available for permeation. Dissolved drug may also be solubilized and partition into a micellar fraction in the presence of solubilizing agents, such as endogenous bile components or formulation additives like polymers and surfactants. When both molecular solubility and solubilization capacity are exceeded, excess drug may separate into a metastable colloidal drug-rich phase (in the presence of polymers) or amorphous drug nanoprecipitates via liquid–liquid phase separation.^24–26^ Drug colloids such as micelles and nanosized drug precipitates have been shown to enhance intestinal absorption and systemic drug exposure of several BCS II compounds.^27–31^ While their small particle size may support faster dissolution, the observed bioavailability improvements often exceed what can be explained by dissolution rate alone. It has been proposed that these colloids enhance mass transport across the intestinal aqueous boundary layer (ABL) adjacent to the epithelial surface, through a mechanism known as the particle drift effect.^29,32,33^ The nanosized colloidal particles can penetrate the ABL via Brownian motion and settle adjacent to the epithelium. This increases the local dissolution rate and thus the free concentration at the epithelium, thereby enhancing drug flux (*J*) according to Fick’s first law of mass transport equation,

$$J=-D\frac{dC}{dx}$$ 1

where the diffusion flux is dependent on the diffusion coefficient (*D*), the concentration gradient of the diffusing species (*C*) and the distance through which the solute moves (*x*).

**Figure 1:**
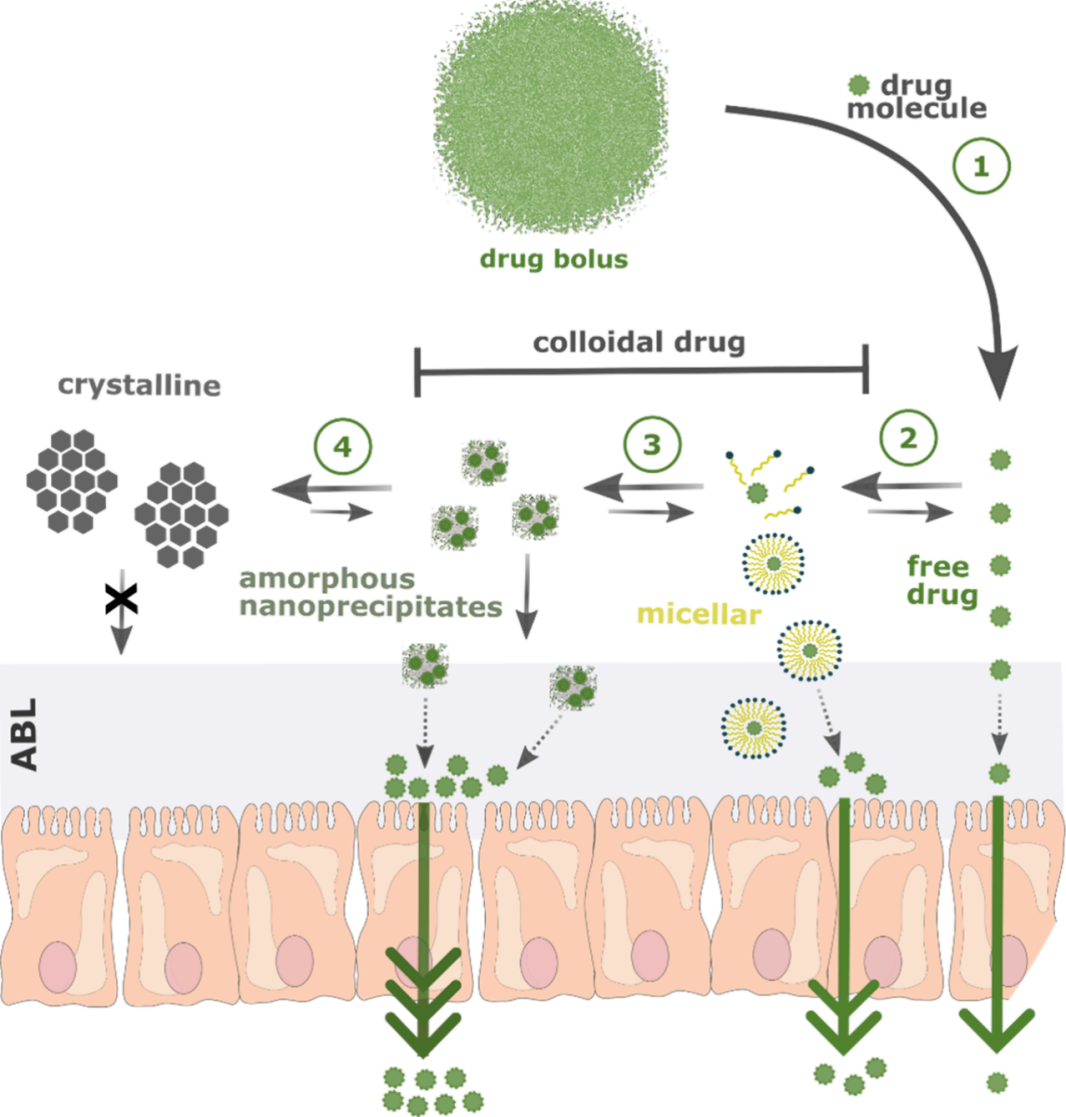
Solubility-permeability interplay of poorly water-soluble drugs, such as the majority of PROTACs. When a drug bolus of the formulation is emptied into the intestinal lumen (1) free drug dissolves up to its maximum free solute activity, which is the amorphous solubility for amorphous drugs. (2) In the presence of solubilizing agents (bile, excipients), the drug can be solubilized until the solubilizing capacity of the solubilizing agents is saturated. (3) Drug that is in excess with respect to amorphous solubility will then precipitate into amorphous nanoprecipitates or drug-rich colloids (typically in the presence of polymer) through liquid–liquid phase separation, which (4) often triggers immediate crystallization of the drug. These mechanisms do not proceed in a strictly sequential manner, since the drug can transition directly between species as it dissolves. The quantitative transport across the intestinal aqueous boundary layer (ABL) and the epithelium is dependent on the drug species present in the intestinal lumen.

Although the particle drift effect has been widely studied in association with various colloidal drug species, mechanistic and quantitative in vivo insights into the interplay between solubility, dissolution, solubilization, precipitation, ABL transport, and permeability remain sparse. Current literature often (1) lacks in vivo data, (2) lacks physicochemical characterization of the in vivo generated drug species, and (3) focuses on BCS Class II drugs that are not representative of PROTACs. In vivo data on the impact of colloidal species to increase fraction dose absorbed and oral bioavailability of PROTACs are essential to facilitate the selection of optimal formulation strategies for this emerging modality.

The main objective of this mechanistic PROTAC study was to determine the effect of colloidal species, in particular amorphous drug nanoprecipitates, on the intestinal absorption of ARV-110 (bavdeglutamide) and ARV-471 (vepdegestrant) based on systemic plasma exposure after intraduodenal administration at ascending doses to rats. In vivo data were complemented with in vitro data on formulation properties, amorphous and crystalline solubility in relevant media, solubilization dynamics, and characterization of emerging drug particles. Finally, the direct effect of in situ-formed amorphous drug nanoprecipitates was directly determined in vivo by dosing two intraduodenal amorphous formulations with identical composition and free solubility, where one contained amorphous drug nanoprecipitates, and the other did not.

## 2 Materials and Methods

### 2.1 Study Drugs and Other Chemicals

The study drugs ARV-110 (purity >98%) and ARV-471 (purity >95.4%) were purchased from Excenen PharmaTech Co., Ltd. (Shanghai, China). Glyceryl monooleate, sodium taurocholate, HPLC grade dimethyl sulfoxide (DMSO), sodium hydroxide (NaOH), d-α-Tocopherol polyethylene glycol 1000 succinate (TPGS), sodium chloride (NaCl), and potassium dihydrogen phosphate (KH_2_PO_4_) were supplied by Sigma-Aldrich (St. Louis, MO, USA). Maleic acid was obtained from Thermo Fisher Scientific (Waltham, MA, USA). Lecithin, sodium oleate, and sodium chenodeoxycholic acid were purchased from TCI Europe (Zwijndrecht, Belgium). Simulated rat intestinal fluid (ratSIF) was prepared according to the protocol by Berghausen et al.^34^ Fed state and fasted state simulated intestinal fluid V1 (FeSSIF and FaSSIF-V1) was prepared as per the instructions of the manufacturer. FeSSIF buffer concentrate and 3F powder were procured from biorelevant.com Ltd. (Croydon, UK).

### 2.2 Physicochemical Characterization

#### 2.2.1 Solubility of ARV-110 and ARV-471 in Aqueous Media

The solubilities of ARV-110 and ARV-471 were determined in triplicate in phosphate buffer (50 mM, pH 6.8), FaSSIF-V1 (pH 6.8), FeSSIF (pH 5.0) and ratSIF (pH 6.0). Both crystalline (thermodynamic) and amorphous (kinetic) aqueous solubility were determined for ARV-110. Given that ARV-471 was obtained already in amorphous form, only the amorphous solubility was tested. All solubility values were considered apparent, i.e., including the concentration of both molecularly dissolved drug and drug solubilized by media components (micelle-bound). The solubilization factor (*f*) in ratSIF was determined as the ratio of the amorphous solubilities in phosphate buffer and ratSIF.

Crystalline equilibrium solubility was determined by adding an excess of crystalline drug powder (500 μg of ARV-110) to a 10 mL glass vial with the respective medium (37 °C, 100 rpm stirring). A volume of 100 μL was sampled after 24 h followed by centrifugation (12,100*g*, 10 min) (MiniSpin, Eppendorf, Hamburg, Germany). 50 μL of the supernatant was prepared for LC–MS/MS analysis.

Amorphous solubility in different media was determined using an ultracentrifugation assay: 25 μL of a highly concentrated drug solution (ARV-110:3.0 mg/mL; ARV-471:25.0 mg/mL) in DMSO was added to 2.5 mL of the respective medium and vortexed thoroughly. The high excess of drug relative to the expected amorphous solubility was used to presaturate the polypropylene tube surface and mitigate the binding to the centrifugation tubes. The drug-spiked medium was then transferred into polypropylene centrifugation tubes, equilibrated for 5 min, and centrifuged at 194,000*g* for 35 min using an XPN-100 ultracentrifuge (Beckmann Coulter Inc., Brea, CA, USA) with a 50.2 Ti rotor to pellet the nanocolloidal structures as well as larger drug particles. 100 μL of the supernatant was immediately diluted 2–50-fold in methanol and subsequently prepared for LC–MS/MS analysis.

#### 2.2.2 Solubilization and Precipitation Propensity of Drugs in the Vehicle and the Rat Intestine

The dissolved and solubilized fraction of the drug (apparent solubility) vs the drug fraction precipitated via LLPS into amorphous drug nanoprecipitates or in larger noncolloidal precipitates was determined using an ultracentrifugation assay. All in vivo administered doses and concentrations were tested in the freshly prepared vehicle, as well as diluted with ratSIF to simulate in vivo conditions and estimate the dissolution and precipitation dynamics directly after bolus injection of the vehicle into the rat duodenum.

For determinations in the vehicle, the suspensions were prepared as described in Section 2.3.2 and subjected to the same ultracentrifugation conditions as described in Section 2.2.1. To estimate the fraction of the drug precipitated and dissolved in the rat intestine after intraduodenal dosing, 1 mL of ratSIF was added to 2 mL of the vehicle. This mixture was vortexed and thereafter ultracentrifuged. A ratio of 2 parts of vehicle to 1 part of ratSIF was based on the assumption that an average bolus injection volume of 500 μL per rat is diluted with an estimated 200–300 μL of duodenal fluids in the fasted rat.^35–37^ After a 20–500-fold dilution of the supernatant in methanol, the samples were prepared and analyzed with the LC–MS/MS assay. The fraction of molecularly dissolved drug was estimated from the micellar drug concentration multiplied by the solubilization factor in ratSIF.

#### 2.2.3 Characterization of Drug Precipitates

Drug precipitates emerging in the vehicle after preparation were characterized with respect to particle size, size distribution, and solid-state properties to mechanistically evaluate their impact on intestinal absorption. The particle size of the colloidal species generated upon preparing the drug vehicle by solvent shifting or simple powder addition was determined by dynamic light scattering (DLS) using a Nanotrac Wave (Microtrac Inc., USA). A sample of the vehicle at each concentration was analyzed immediately after preparation (*t*_0_) and after 10 min (*t*_10_), as this was the maximum time between vehicle preparation and intraduodenal dosing. A measurement of the blank vehicle was used to adjust for the signal originating from TPGS micelles or other colloidal species. A polydispersity index (PDI) was moreover determined to assess the size uniformity of the amorphous nanoprecipitates. To characterize structural aspects of the emerging precipitates, wide-angle X-ray scattering (WAXS) was performed using a Xeuss QXoom 2.0 instrument (Xenocs, Grenoble, France) equipped with a microfocus Cu Kα X-ray source (λ = 1.54 Å) operated at 50 kV, 0.6 mA and a Pilatus 300 R detector (Dectris, Baden-Dättwil, Switzerland). The scattering patterns were recorded in the *q*-range of 1.2–3.1 Å^–1^ (2θ range 15–45°). Each measurement was done over 1 h per sample and repeated 5 times to monitor potential temporal structure changes of the sample over 6 h. Samples of the emerging precipitates were obtained by ultracentrifugation (see Section 2.2.1) of the highest dose (1.0 mg/kg for ARV-110 and 5.0 mg/kg for ARV-471). The sedimented precipitate was scraped off the centrifugation tubes and mounted on a sample holder with 50 μm-thick kapton windows to avoid exposure of the sample to the vacuum. The background scattering of the instrument was measured with an empty holder and subtracted from each sample. Crystalline bulk powder of ARV-110 was measured in the same way as a reference for crystallinity. Since the bulk powder of ARV-471 was already in an amorphous state, a crystalline reference diffractogram could not be obtained for ARV-471. Polarized light microscopy was applied to verify the solid state of the precipitates and to estimate the size of larger particles beyond the nanometer size range measurable with DLS. These tests were carried out with an Olympus BX51 microscope (Olympus, Tokyo, Japan) equipped with an UMPlanFI 10x lens and an Olympus U-POT polarizer.

### 2.3 Pharmacokinetic Studies in Rats

This animal study was approved by the local ethics committee for animal research (5.8.18-00250/2022) in Uppsala, Sweden. A total of 45 male Sprague–Dawley rats (NTac) from Taconic (Ejby, Denmark) with body weights ranging from 240 to 350 g (aged 6–12 weeks) were used for 8 study groups (5–6 per group). All animals were allowed to acclimatize for at least 1 week in the Animal Department before the start of the experiment. They were allowed water and food ad libitum until initiation of fasting. Housing conditions were 21–22 °C at a 12–12 h light–dark cycle. Animals were evaluated for general welfare twice daily by the Animal Department staff.

All rats receiving an intraduodenal bolus administration were fasted for 14 h before the start of the experiment to ensure consistent intestinal luminal conditions. On the study day, the rats were anesthetized using an intraperitoneal injection of a 10% w/v inactin (thiobutabarbital) solution (120 mg/kg). The total study duration was approximately 7 h (1 h for surgery and 6 h for sampling), during which the rats were continuously sedated. Body temperature was maintained at 37.5 ± 0.5 °C by an infrared lamp and a temperature regulator controlling a heating pad placed below the rat. A cannula was inserted into the trachea to ensure unobstructed breathing during the procedure and the femoral artery and vein were cannulated with a PE-50 polyethylene catheter. Systemic arterial blood pressure was continuously recorded by connecting an arterial catheter containing 20 IU/mL heparin isotonic saline to a transducer operating a PowerLab system (AD Instruments, UK). Animals with a mean arterial blood pressure <70 mmHgwere excluded from the study. The animals were euthanized with a potassium chloride injection into the heart at study termination.

#### 2.3.1 Intraduodenal Bolus Studies

Intraduodenal bolus dosing was employed to mechanistically evaluate the absorption kinetics of ARV-110 and ARV-471 in the small intestine, omitting gastric emptying to minimize variability in absorption kinetics. A polyethylene tube (ID: 0.76 mm; OD: 1.22 mm) attached to a syringe containing the study formulation was guided with a larger tube through the mouth and stomach directly into the duodenum, about 2 cm distal to the pyloric sphincter. The syringe was prepared with 2 mL of the respective drug formulation per kilogram of rat body weight and another 0.11 mL to account for the dead volume of the inner tube. The drug formulation was administered as a single bolus directly into the duodenum. The correct position of the administration tube in the proximal duodenum as well as the complete delivery and absence of reflux back into the gastric compartment, was confirmed by visual inspection of the small intestine and stomach during and within the first 2 min after injection. Blood samples (300 μL) were drawn from the femoral artery at 10, 20, 40, 60, 90, 120, 240, and 360 min and transferred into a lithium heparin-prepared tube. After every second blood sampling, the withdrawn volume was replaced by injecting 600 μL saline with 70 mg/mL bovine serum albumin through the femoral artery. The detailed surgical procedure and protocol for the intraduodenal bolus study are described in a previous study.^18^ Despite the known serin protease-mediated degradation of PROTACs, no inhibitor of extracellular proteases was added in this study to avoid disrupting the physiological homeostasis of the intestinal lumen.^18,38^

#### 2.3.2 Study Formulations

For the in vivo pharmacokinetic studies, the PROTACs were administered as nanosuspensions with the same vehicle composition but at different doses. ARV-110 was dosed at 0.04, 0.2, and 1.0 mg/kg, corresponding to drug concentrations in the vehicle of 0.02, 0.1, and 0.5 mg/mL. ARV-471 was dosed at 0.2, 1.0, 2.5, and 5.0 mg/kg (0.1, 0.5, 1.25, and 2.5 mg/mL). For these seven cohorts, the vehicle was prepared by spiking phosphate buffer (50 mM, pH 6.8) containing 0.05% TPGS with a highly concentrated DMSO stock solution to create amorphous colloidal particles with varying particle sizes. In an eighth cohort, ARV-471 was again dosed at 1.0 mg/kg (0.5 mg/mL), with the same vehicle composition but a different vehicle preparation method than for the amorphous colloidal particles; this preparation is hereafter referred to as the amorphous powder suspension. Here, the blank vehicle was prepared separately, and the amorphous ARV-471 powder was suspended in the vehicle to create an amorphous suspension without a nanocolloidal amorphous phase. The vehicle of all study groups consisted of 10% DMSO, in which the drug was initially dissolved, and 90% phosphate buffer (50 mM, pH 6.8) containing 0.05% TPGS. All drug suspensions were freshly prepared within 10 min prior to intraduodenal injection.

### 2.4 Plasma Pharmacokinetic Analysis

Descriptive pharmacokinetic (PK) parameters were calculated through noncompartmental analysis of plasma concentration–time data following *i.v*. (data from a previous study performed in our laboratory^39^) and intraduodenal bolus administrations. Peak plasma concentrations (*C*_max_) and the time to *C*_max_ (*t*_max_) were read directly from the plasma concentration–time data for each rat. The area under the plasma concentration–time curve from 0 to 6 h (AUC_0–6h_) was computed employing the linear trapezoidal method (lin up log down). The 0–6 h bioavailability (*F*_0–6h_) from the intraduodenally administered formulations of ARV-110 and ARV-471 was calculated by dividing AUC_0–6h_ following intraduodenal administration (AUC_0–6h;ID_) by the area under the curve following an IV bolus (AUC_0–6h;IV_), corrected by the respective dose (eq 2).

$$F_{0-6h}(\%)=100\%\times (\frac{{\mathrm{AUC}}_{0-6h;\mathrm{ID}}}{{\mathrm{AUC}}_{0-6h;\mathrm{IV}}})\times \left(\frac{{\mathrm{Dose}}_{\mathrm{IV}}}{{\mathrm{Dose}}_{\mathrm{ID}}}\right)$$ 2

Numerical deconvolution analysis was employed as originally described by Langenbucher et al. to estimate the input function, i.e., the rate and extent of drug absorption of ARV-110 and ARV-471 into the central compartment based on experimental plasma concentration–time data in rats.^40^ A known impulse-response function, i.e., observed data after i.v. bolus administration was used to describe the drug disposition. A series of discrete input impulses, each followed by the corresponding response function, is superimposed to best fit the observed plasma data after intraduodenal dosing. An input rate (mass time^–1^) over time for each subject can be constructed by superimposing many such impulses with an impulse-response curve. Temporal resolution of the individual impulses was set at 1.2 min, and the integration of this input function provided a cumulative amount absorbed, which, related to the total dose, is denoted as the cumulative fraction absorbed (*f*_abs_). Deconvolution was performed in R (version 4.2.1) using RStudio (version 202.7.1), utilizing the *Rivivc* package for deconvolution and the *tidyr* package for data wrangling.

To mechanistically evaluate the solubility-permeability interplay of the PROTACs, the in vivo absorptive flux (*J*_0–0.5h_) within the first 0.5 h of the experiments was calculated from the deconvoluted input rate. Within the 0–0.5 h interval, the in vivo conditions, including the injected dose volume, particle diameter, and the distribution of the drug into different drug fractions, were well-characterized and assumed to be stable, allowing for a mechanistic evaluation of the factors contributing to PROTAC absorption.

### 2.5 Bioanalysis Using LC–MS/MS

The PROTACs ARV-110 and ARV-471 were analyzed in rat plasma or aqueous solutions using an ACQUITY-UPLC I-Class system (Waters, Manchester, UK) coupled to a Xevo-TQSmicro tandem quadrupole mass spectrometer (Waters, Manchester, UK) via an ESI interface operated in positive ionization mode. The analytes as well as the chosen internal standards (IS) warfarin (for ARV-110) and verapamil (for ARV-471) were separated from the plasma matrix with a BEH C18 column (particle diameter 1.7 μm, diameter x length 2.1 × 50 mm) attached to a BEH C18 VanGuard precolumn (1.7 μm, 2.1 × 5 mm) (Waters, Manchester, UK) held at 40 °C with a gradient of mobile phase (A) consisting of 0.1% FA in water and (B) 0.1% FA in acetonitrile over 4.0 min, as described a previous study.^41^ The injection volume was set to 5 μL and the samples were run at a flow rate of 0.3 mL/min. Quantitative analysis was performed with tandem mass spectrometry using selective-reaction monitoring (SRM) in positive ionization mode at the respective transitions from the [M + H]^+^ ions to their daughter ions at *m*/*z* 812.6 → 452.3 (ARV-110) and *m*/*z* 724.68 → 396.39 (ARV-471). Calibration samples were prepared at 0.5, 2, 5, 10, 50, 200, and 1000 ng/mL, with quality control samples prepared separately at 0.5, 1.5, 150, and 750 ng/mL. Detailed information on the bioanalytical method and sample preparation for the quantification of ARV-110 and ARV-471 in rat plasma, including a full validation according to FDA and EMA guidelines, is described in a previous study.^41^

The same analytical method with differing sample pretreatments and a calibration range of 2 ng/mL to 200 ng/mL and quality controls at 2, 5, 50, and 150 ng/mL were used for the analysis of ARV-110 and ARV-471 in aqueous solutions, i.e. simulated intestinal fluids, phosphate buffer, or the vehicle for intraduodenal dosing. 50 μL of sample was added to 440 μL of a solvent mixture consisting of acetonitrile, water, and methanol at a ratio of 63:30:7 (v/v/v), and 10 μL of internal standard solution (warfarin 37.5 ng/mL; verapamil 10 ng/mL) was directly added to amber glass vials following an injection of 5 μL of the diluted sample into the LC–MS/MS system.

### 2.6 Statistical Analysis

Descriptive pharmacokinetic parameters of the ARV-471 vehicle with nanocolloidal amorphous particles were statistically tested against the drug vehicle with suspended amorphous powder by employing a parametric, unpaired *t* test. A *p*-value < 0.05 was considered statistically significant. Data were expressed as individual values, median (range), and mean ± standard deviation (SD) or standard error of the mean (SEM). Dose proportionality of pharmacokinetic parameters (*C*_max_, AUC_6h_) was assessed using the power model:

ln(PKparameter)=α+β×ln(Dose)+ε 3

Linear regression was performed on log-transformed PK parameters versus dose. Point estimates and 90% confidence intervals (CIs) for β were obtained. Dose linearity was considered if the 90% CI for β included 1.0. Additionally, graphical assessment was performed by plotting log-transformed PK parameters against log-transformed dose, with the fitted regression line overlaid. Deviations of β from unity were interpreted as less than dose-proportional (β < 1) or more than dose-proportional (β > 1) increases in exposure with dose escalation.

## 3 Results

### 3.1 Dose Escalation of ARV-110 and ARV-471 in the Rat

The plasma concentration–time profiles after single intraduodenal dosing of ARV-110 (0.04–1.0 mg/kg) and ARV-471 (0.2–5.0 mg/kg) are shown in Figure 2. The corresponding noncompartmental PK parameters, including i.v. reference data,^18^ are summarized in Table 1 for ARV-110 and Table 2 for ARV-471.

**Figure 2:**
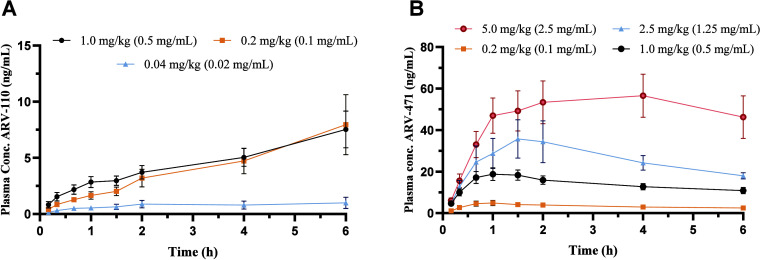
Plasma concentration–time profiles after intraduodenal bolus administration at ascending single doses for ARV-110 (A) and ARV-471 (B) in the rat (*n* = 5–6). Data is presented as mean ± standard error of the mean (SEM).

**Table 1:** Mean ± SD or Median (Range) Noncompartmental Pharmacokinetic Parameters Following Intravenous (i.v.) and Intraduodenal Administrations of ARV-110 at Ascending Doses (*n* = 5–6)^a^,^b^

**PK parameter**	i.v. **bolus**	**intraduodenal bolus**		
	**ARV-110** **(1** mg/kg)	**ARV-110** **(0.04** mg/kg)	**ARV-110** **(0.2** mg/kg)	**ARV-110** **(1.0** mg/kg)
*C*_max_ (ng/mL)		1.4 ± 1.1	7.4 ± 4.8	7.2 ± 3.8
*t*_max_ (h)		6.0 (2.0 – 6.0)	6.0 (4.0 – 6.0)	6.0 (4.0 – 6.0)
AUC_0–6h_ (h·ng/mL)	343.9 ± 26.6	4.5 ± 1.8	23.8 ± 6.7	26.1 ± 5.3
*F*_0–6h_ (%)		32.7 ± 13.1	34.6 ± 1.9	7.6 ± 1.5
*f*_abs_		0.52 ± 0.34	0.53 ± 0.23	0.11 ± 0.07
*J*_0–0.5h_ (μg/min)		0.03 ± 0.02	0.10 ± 0.05	0.10 ± 0.03

a The lower values for *F*_0–6h_ than for *f*_abs_ are not a consequence of first-pass extraction but rather due to the 6 h-sampling period in combination with the long elimination half-life of ARV-110.

b *C*_max_: maximum concentration in plasma. *t*_max_: time to reach *C*_max_. AUC_0–6h_: area under the plasma concentration–time curve between 0 and 6 h. *F*_0–6h_: relative bioavailability between 0 and 6 h based on AUC. *f*_abs_: deconvoluted fraction absorbed. *J*_0–0.5h_: absorptive flux for the initial 0.5 h after dosing.

**Table 2:** Mean ± SD or Median (Range) Noncompartmental Pharmacokinetic (PK) Parameters in Rat Following Single Dose Intravenous (i.v.) and Intraduodenal Administrations of ARV-471 at Ascending Doses (*n* = 5–6)^a^

**PK parameter**	i.v. **bolus**	**intraduodenal bolus**			
	**ARV-471** **(1** mg/kg)	**ARV-471** **(0.2** mg/kg)	**ARV-471** **(1.0** mg/kg)	**ARV-471** **(2.5** mg/kg)	**ARV-471** **(5.0** mg/kg)
*C*_max_ (ng/mL)		5.1 ± 2.9	20.0 ± 6.6	33.5 ± 24.2	69.7 ± 24.2
*t*_max_ (h)		1.0 (0.66–2.0)	1.25 (0.66– 2.0)	2.0 (1.5–4.0)	3.0 (1.0–4.0)
AUC_0–6h_ (h·ng/mL)	557.3 ± 44.1	19.7 ± 3.8	81.8 ± 8.9	151 ± 30.3	286 ± 51.4
*F*_0–6h_ (%)		17.7 ± 3.2	14.5 ± 1.6	10.9 ± 2.2	10.3 ± 1.9
*f*_abs_		0.17 ± 0.08	0.16 ± 0.05	0.12 ± 0.03	0.12 ± 0.03
*J*_0–0.5h_ (μg/min)		0.07 ± 0.03	0.28 ± 0.11	0.43 ± 0.24	0.53 ± 0.23

a *C*_max_: maximum concentration in plasma. *t*_max_: time to reach *C*_max_ in plasma. AUC_0–6h_: area under the plasma concentration–time curve between 0 and 6 h. *F*_0–6h_: relative bioavailability between 0 and 6 h. *f*_abs_: deconvoluted fraction absorbed. *J*_0–0.5h_: absorptive flux for the initial 0.5 h after dosing.

For ARV-110, the *C*_max_, AUC, and *J*_0–0.5h_ increased linearly (Supporting Information Figure S4) between 0.04 and 0.2 mg/kg, resulting in an *f*_abs_ of 0.52 at both doses. At the highest dose of 1.0 mg/kg, no further increases in *C*_max_, AUC, and *J*_0–0.5h_ were observed, resulting in an 80% reduction in *f*_abs_ compared to the lower doses. A dose proportionality analysis (Supporting Information Figure S4) demonstrated a subproportional increase in *C*_max_ and AUC_0–6h_ for ARV-110 with estimated slopes of 0.562 and 0.513 and a 90% confidence interval not covering linearity, i.e., a slope <1.00.

Following single-dose intraduodenal administration of ARV-471, *F*_0–6h_ and *f*_abs_ both decreased modestly from 0.17 at 0.2 mg/kg to 0.12 at 5 mg/kg, respectively. Over the same dose range *t*_max_ was delayed from 1 to 3 h. For the lower doses of 0.2 and 1.0 mg/kg, a dose-proportional increase in *J*_0–0.5h_ was determined, while at higher doses (2.5–5.0 mg/kg) the increase was subproportional. A dose proportionality analysis (Supporting Information Figure S4) demonstrated a less-than-dose-proportional increase in *C*_max_ across the evaluated dose range. AUC_0–6h_ showed a slope of 0.884 (90% CI: 0.788–1.02), with the confidence interval spanning unity, consistent with approximately dose-proportional increases in overall systemic exposure.

### 3.2 Amorphous Drug Nanoprecipitates vs Amorphous Powder of ARV-471 in Rats

The effect of amorphous drug nanoprecipitates on the absorption and bioavailability of ARV-471 was evaluated by comparing a formulation prepared via antisolvent-induced phase separation (yielding nanocolloidal amorphous drug) to an amorphous powder suspension (no nanocolloidal drug particles). Formulations differed only in the physical state of the particles (see [Sec sec3.3] and 3.4). The plasma concentration–time curves are shown in Figure 3A, the temporal resolution of the drug flux into the intestinal epithelium in Figure 3B, and the PK parameters in Table 3.

**Figure 3:**
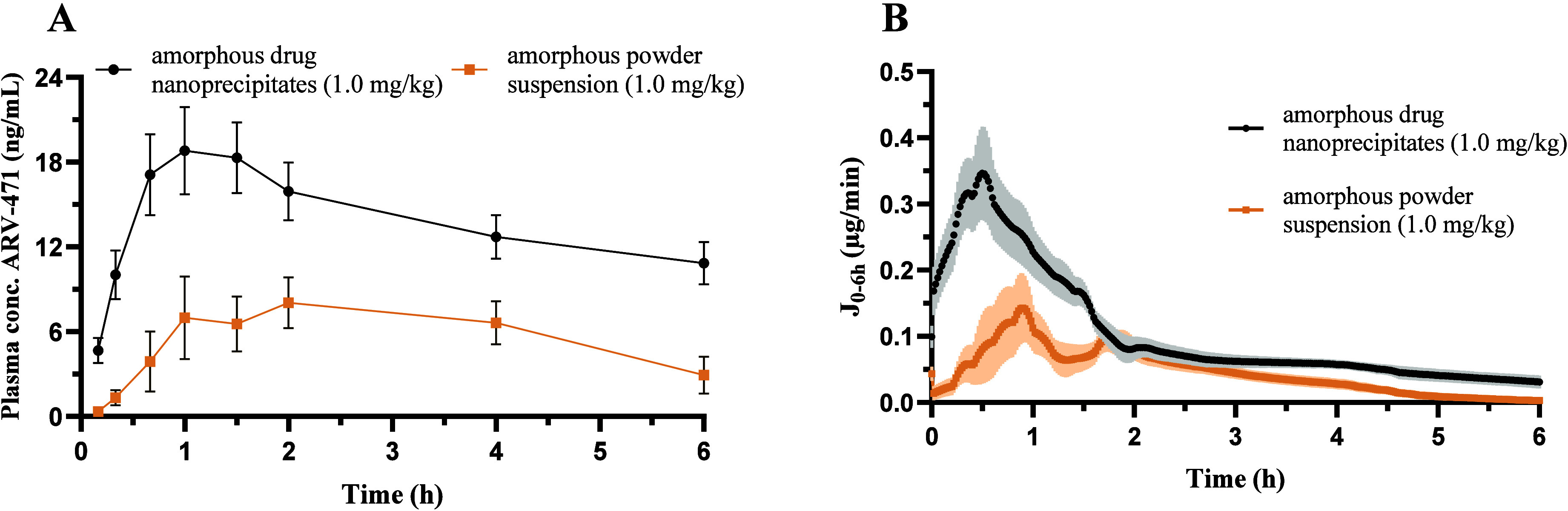
(A) Plasma concentration–time profiles after intraduodenal dosing of ARV-471 at 1.0 mg/kg as a suspension containing amorphous drug nanoprecipitates (●) or suspended amorphous drug powder (■) without any nanocolloidal particles (*n* = 5–6). Data is presented as mean ± standard error of the mean (SEM). (B) Temporal progression of the absorptive flux over 6 h for ARV-471 at 1.0 mg/kg after intraduodenal bolus administration of a vehicle with and without amorphous drug nanoprecipitates. (*n* = 5).

**Table 3:** Mean ± SD or Median Noncompartmental Pharmacokinetic Parameters Following Intraduodenal Administrations of ARV-471 as a Suspension Containing Amorphous Drug Nanoprecipitates and Suspended Powder without any Nanocolloidal Particles (*n* = 5–6)^a^

**PK parameter**	**ARV-471** **(1** mg/kg) **as intraduodenal bolus**	
	**amorphous drug nanoprecipitates**	**amorphous powder suspension**
*C*_max_ (ng/mL)	20.0 ± 6.6*	9.2 ± 5.7
*t*_max_ (h)	1.25 (0.66–2.0)	2.0 (1.0–2.0)
AUC_0–6h_ (h·ng/mL)	81.8 ± 8.9*	34.1 ± 7.4
*F*_0–6h_ (%)	14.5 ± 1.6*	6.1 ± 1.3
*f*_abs_	0.16 ± 0.05**	0.07 ± 0.04
*J*_0–0.5h_ (μg/min)	0.28 ± 0.11***	0.04 ± 0.04

a *C*_max_: maximum concentration in plasma. *t*_max_: time to reach *C*_max_ in plasma. AUC_0‑6h_: area under the plasma concentration–time curve between 0 and 6 h. *F*_0–6h_: relative bioavailability between 0 and 6 h. *f*_abs_: deconvoluted fraction absorbed. *J*_0–0.5h_: absorptive flux for the initial 0.5 h after dosing. Statistics were conducted by an unpaired *t* test. Significance between the study groups is indicated by *, with *p < 0.05, **p < 0.01, and ***p < 0.001.

The intraluminal formation of amorphous drug nanoprecipitates of ARV-471 resulted in a > 2-fold increase in *C*_max_, a shorter time to reach *C*_max_, and a 2.4-fold (*p* = 0.016) increased AUC_0–6h_ of ARV-471. In the initial absorption phase (0–0.5 h), where the dynamics of drug dissolution were well-characterized, the in vivo flux of the vehicle containing amorphous drug nanoprecipitates was 7-fold higher (*p* = 0.0010). This is also highlighted in Figure 3B, showing a pronounced difference in absorptive flux within the first 1.5 h after dosing, whereafter the flux of the two formulations converges.

### 3.3 Characterization of Precipitates and Colloidal Drug Species upon Supersaturation of ARV-110 and ARV-471

As depicted in Figure 4, the average hydrodynamic ARV-110 particle diameter of the precipitates 10 min after preparation increased in the nanometer range with dose from 44.1 ± 2.4 nm (0.04 mg/kg), to 64.6 ± 16.7 nm (0.2 mg/kg), and 116.7 ± 25.3 nm (1.0 mg/kg).

**Figure 4:**
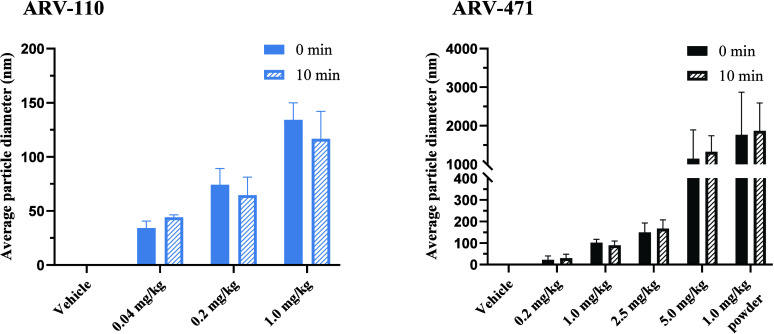
Average (±SD) hydrodynamic diameter of ARV-110 (left) and ARV-471 (right) particles in the vehicle at different doses. The suspensions were tested in triplicate right after preparation and after 10 min. All groups correspond to the ascending doses of the vehicle prepared by solvent shifting. For ARV-471, the 1.0 mg/kg powder group was prepared by suspending amorphous powder in the vehicle.

The ARV-471 particle diameter also increased with dose from 30.0 ± 15.2 nm (0.2 mg/kg) to 89.9 ± 19.2 nm (1.0 mg/kg) to 166.5 ± 40.9 nm (2.5 mg/kg) up to micron-sized precipitates of 1326 ± 451 nm (5.0 mg/kg). No substantial growth of particles over 10 min was observed at any dose. The vehicle containing suspended amorphous powder exhibited distinctly larger particles, with an average diameter of 1765 ± 1105 nm 10 min after vehicle preparation, compared to the corresponding formulation prepared via antisolvent-induced generation of amorphous drug nanoprecipitates (89.9 ± 19.2 nm). Across all doses, the particle size distribution remained narrow 10 min after formulation preparation, as reflected by PDI values <0.3 (Supporting Information Table S1). A more detailed distribution of the particle sizes at respective doses can be found in the Supporting Information Figures S2 and S3.

As shown in Figure 5, the antisolvent-induced colloidal drug phase showed no evidence of diffractogram peaks, indicative of the amorphous state of the precipitates. None of the diffractograms showed distinct changes or any peaks over 6 h of measurements, suggesting that the PROTACs were kinetically stable in their amorphous state. The findings were further verified by polarized light microscopy (PLM) of the sedimented precipitates, both in the vehicle as well as after diluting with ratSIF to mimic in vivo conditions. The resulting images can be seen in the Supporting Information Figure S1, exposing no clear birefringence in the vehicle or vehicle diluted with ratSIF, underlining the amorphous nature of the precipitates. To further assess phase behavior under hydrated conditions, the wet glass transition temperature (*T*_g_) of ARV-110 and ARV-471 was determined. For both PROTACs, a wet *T*_g_ of approximately 70 °C was observed (data not shown), well above physiological temperature (∼37 °C), indicating that the amorphous precipitates remain in a glassy, rather than liquid-like, state under in vivo conditions.

**Figure 5:**
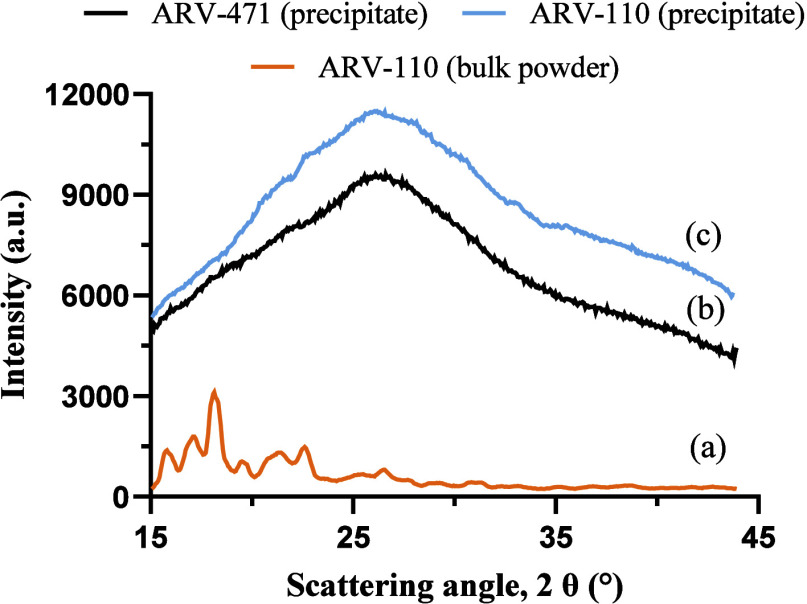
X-ray diffractograms of the crystalline bulk powder of ARV-110 (a) in comparison to the *in situ* generated precipitates of ARV-471 (b) and ARV-110 (c).

### 3.4 Extent of Solubilization and Precipitation of Drugs in Vehicle and Rat Intestinal Fluids

As shown in Table 4, the PROTACs predominantly precipitated into their undissolved amorphous form across all administered doses, except for ARV-471 at the lowest dose of 0.2 mg/kg, where most of the drug (81.5%) was dissolved and/or solubilized. The estimated free drug concentrations based on the solubilization factor in the vehicle:ratSIF medium ranged from 0.9–1.2 μg/mL for ARV-110 and 2.0–2.5 μg/mL for ARV-471. Thus, the vast majority (>95.5%) of the PROTACs remained in colloidal form or as larger precipitates at all tested doses pre- and postdosing. The reduced dissolved and micelle-bound concentrations observed at the highest doses of ARV-110 (1.0 mg/kg) and ARV-471 (5.0 mg/kg) likely reflect saturation of the solubilization capacity of TPGS in the vehicle. In contrast, this concentration drop was not observed in the vehicle:ratSIF medium, where concentrations remained consistent across the dose range, likely due to the higher levels of solubilizing components present in ratSIF.

**Table 4:** Mean Concentration (±SD) of the Dissolved and Micelle-Bound Drug, i.e., the Apparent Amorphous Solubility, and the Precipitated Drug Sedimented during Ultracentrifugation^a^

**nominal vehicle concentration** **(in vivo dose)**	**vehicle**		**vehicle:ratSIF (2:1)**	
	**dissolved and micelle-bound drug**(μg/mL)	**precipitated drug**(μg/mL)	**dissolved and micelle-bound drug**(μg/mL)	**precipitated drug**(μg/mL)
**ARV-110**				
0.02 mg/mL (0.04 mg/kg)	8.2 ± 0.9	11.8 ± 0.9	4.3 ± 1.4	9.1 ± 1.4
0.1 mg/mL (0.2 mg/kg)	8.1 ± 1.0	91.9 ± 1.0	4.7 ± 0.3	62.0 ± 0.3
0.5 mg/mL (1.0 mg/kg)	3.3 ± 0.3	497 ± 0.3	5.6 ± 1.5	328 ± 1.5
**ARV-471**				
0.1 mg/mL (0.2 mg/kg)	81.5 ± 4.9	18.5 ± 4.9	43.7 ± 2.5	23.0 ± 2.5
0.5 mg/mL (1.0 mg/kg)	88.7 ± 8.3	411 ± 8.3	47.1 ± 2.0	286 ± 2.0
1.25 mg/mL (2.5 mg/kg)	55.3 ± 6.3	1190 ± 6.3	35.8 ± 3.5	798 ± 3.5
2.5 mg/mL (5.0 mg/kg)	15.7 ± 5.2	2480 ± 5.2	39.6 ± 5.3	1630 ± 5.3
0.5 mg/mL (powder) (1.0 mg/kg)	76.2 ± 7.2	424 ± 7.2	44.3 ± 1.8	289 ± 1.8

a Measurements were done in the vehicle and in the vehicle diluted with ratSIF (2:1) to simulate in vivo conditions after intraduodenal injections. (*n* = 3) Due to the dilution with ratSIF, the target drug concentration in vehicle:ratSIF was at two-thirds of the nominal vehicle concentration.

### 3.5 Solubility of ARV-110 and ARV-471 in Different Media

As summarized in Table 5, ARV-110 and ARV-471 showed poor solubility in phosphate buffer and benefited from the presence of solubilizing agents in FaSSIF, ratSIF, and FeSSIF by a marked increase in apparent solubility. The solubilization factor (f) - ratio of the amorphous solubilities in ratSIF and phosphate buffer - was 4.7 for ARV-110 and 18.4 for ARV-471. A 24-fold (ARV-110) and 61-fold (ARV-471) increase in apparent solubility in FeSSIF compared to phosphate buffer was likely attributable to solubilization and a higher degree of ionization of the basic PROTACs at the lower pH of pH 5.0 in FeSSIF. Moreover, the amorphous solubility advantage of ARV-110, *i.e*., the extent of supersaturation achievable before colloidal separation occurs, was 64-fold in buffer, highlighting the importance of this metastable equilibrium for poorly soluble drugs such as ARV-110.

**Table 5:** Mean (±SD) Solubility of ARV-110 and ARV-471 in Different Media^a^

**medium**	**ARV-110**		**ARV-471**	
	**crystalline solubility (μg/mL)**	**amorphous solubility**(μg/mL)	**crystalline solubility**(μg/mL)	**amorphous solubility**(μg/mL)
phosphate buffer pH 6.8	0.012 ± 0.005	0.77 ± 0.18		3.26 ± 0.52
FaSSIF pH 6.8	0.195 ± 0.050	1.23 ± 0.10		19.9 ± 0.58
ratSIF pH 6.0	1.02 ± 0.302	3.60 ± 0.40		60.1 ± 3.50
FeSSIF pH 5.0		18.5 ± 2.22		199 ± 32.1

a Amorphous solubilities were determined by an antisolvent-shift method followed by ultracentrifugation. ARV-110 crystalline solubility was determined by a shake-flask method.

## 4 Discussion

This in vivo rat study determined the impact of amorphous drug nanoprecipitates on the intestinal absorption of two PROTACs, ARV-110 and ARV-471. These PROTACs are characterized by very poor aqueous solubility, low to intermediate intestinal permeability, and sensitivity toward enzymatic degradation by extracellular proteases in the rat intestinal lumen.^18,42^ In this study, both PROTACs predominantly emerged as stable amorphous drug nanoparticles of varying sizes in the intestinal lumen after dosing. The presence of these drug species played a key role in enhancing the intestinal absorption of both PROTACs beyond the amorphous solubility threshold, with a central impact for formulation development strategies.

### 4.1 Dose Escalation Study in Rats

At all doses in this rat study (0.04 – 5.0 mg/kg), the amorphous solubility for both ARV-110 (4.3 μg/mL) and ARV-471 (43.7 μg/mL) was exceeded at least 2-fold. This led to the precipitation of amorphous drug nanoparticles at all dose levels and larger amorphous aggregates at the highest dose of ARV-471 (5 mg/kg). According to the original theory of intestinal drug absorption, the drug flux across the epithelium should plateau once the solubility limit is reached, as the molecularly dissolved fraction, as the main driving force for permeation, reaches a pseudo steady state.^43–45^ However, when evaluating dose-proportionality, *J*_0–0.5h_, *C*_max,_ and AUC_0–6h_ increased linearly up to a dose level at which the amorphous solubility was exceeded 14-fold for ARV-110 (0.2 mg/kg) and 12-fold for ARV-471 (1.0 mg/kg). The proportional increase in flux well beyond the amorphous solubility was likely a result of the formation of colloidal amorphous precipitates in the rat lumen, as the molecularly dissolved and micelle-bound drug concentrations were the same across the entire dose range for each PROTAC.

The potential of nanoparticles and colloidal species to enhance intestinal absorption and plasma exposure has been reported in vitro^46–50^ and in vivo.^27,29,32,51^ Increased absorption is proposed to result from the ability of drug micelles and colloidal drug particles to diffuse across the ABL and serve as a drug reservoir, thereby enhancing dissolution rate and elevating local free drug concentration directly at the epithelial surface.^27,31,32,49,52–58^ This mechanism, termed the “particle drifting effect”,^33^ provided a plausible explanation for the observed pharmacokinetics of ARV-110 and ARV-471. At lower doses, the small hydrodynamic dimensions (≤90 nm) of the amorphous drug nanoprecipitates allowed them to diffuse like molecular solutes across the ABL, as they do not rely on convective transport at this size.^59^ Once at the membrane surface, the amorphous drug nanoprecipitates readily dissolved and sustained a local supersaturation. By this mechanism, an absorptive flux advantage via particle drifting, as highlighted by the green area in Figure 6A+B, can be achieved until a certain particle concentration is reached. The extent of this flux advantage is dependent on the solubility of the drug, its solid-state properties, and the particle concentration, which also dictated the formation and size of the precipitates.

**Figure 6:**
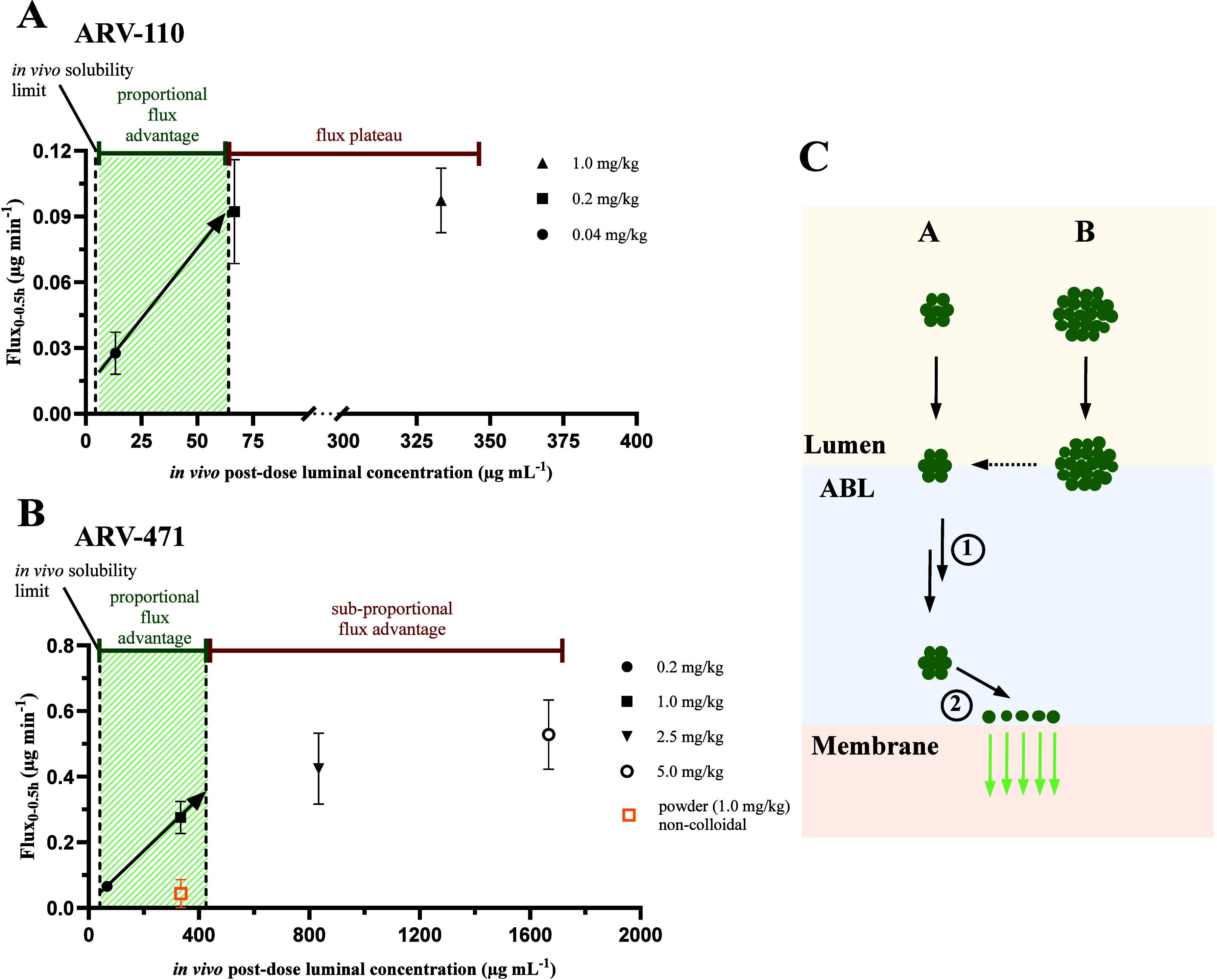
Mean absorptive flux ± SEM of ARV-110 (A) and ARV-471 (B) versus luminal drug bulk concentrations at ascending doses. (*n* = 5–6) The first dashed line represents the concentration at which the solubility in rat intestinal fluids is saturated, based on solubility studies of the formulations in ratSIF. Up to this concentration, absorptive flux is only driven by the free drug monomer and micellar drug.^31^ The second dashed line represents the approximated maximum concentration to which flux increased dose proportionally. The green area represents the gained flux advantage through the presence of amorphous drug nanoprecipitates. The orange square highlights the reduced flux of the noncolloidal formulation of ARV-471. Panel C depicts the different diffusion pathways of nanocolloidal drug particles through the aqueous boundary layer (ABL), explaining the absorption limitations of ARV-110 and ARV-471 at increasing particle concentration. While ARV-471 absorption was limited by efficient particle drift into the ABL due to size limitations (1), absorption of ARV-110 was likely limited by the dissolution of the nanoprecipitates at the epithelial surface due to solubility saturation (2).

If the linear increase in flux for ARV-110 and ARV-471 at lower doses can be attributed to the particle-drift effect, what then explains their different behavior at higher doses? For ARV-110, flux plateaued at 0.2 mg/kg, whereas for ARV-471, the data suggest an increased flux up to 5 mg/kg, but at a reduced rate. Since the flux plateau occurred at ARV-110 precipitate sizes below 100 nm, it was unlikely that the diffusivity of the colloids limited flux. Rather, the plateau reflected saturation of the intestinal ABL and of the epithelial membrane as a result of the very low amorphous solubility of ARV-110. This means that solubility became rate-limiting, and additional drug particles could not further enhance local dissolution and flux. Similar in vitro findings are reported for BCS class II drugs, where amorphous solubility rather than particle size limits further increases in drug flux from colloidal drug species.^49,53^

For ARV-471, on the other hand, the intestinal absorption did not plateau but increased subproportionally with dose even when the amorphous solubility was exceeded by up to 40-fold, suggesting that solubility was not rate-limiting for absorptive flux. Instead, at higher particle concentrations, the dimensions of the emerging amorphous precipitates increased beyond 100 nm and up to the μm size range. At this size, particle drifting across the ABL diminished, as convective transport drastically decreased across the ABL. This is illustrated in Figure 6C (path B), where higher drug concentrations can lead to the formation of large particles, which cannot diffuse as efficiently across the ABL. These larger particles either travel at a slower rate or must first dissolve before they can contribute to an increased absorption.^26^ The absorption enhancement for ARV-471 then became ABL diffusion rate-limited. These findings are corroborated by Kesisoglou et al., who identified a particle size threshold of 102 nm for anacetrapib (also BCS class IV). Above this size, *C*_max_ and AUC were reduced by more than 50% in dogs.^27^ It should be noted that despite the subproportional increase in flux from 1.0 mg/kg, the total plasma exposure for ARV-471 remained near dose-proportional up to 5 mg/kg. This is because larger amorphous drug precipitates remained stable and could act as a rapidly dissolving luminal drug reservoir during intestinal transit, which was reflected in the increased *t*_max_ from 1.0 h at 0.2 mg/kg to 3.0 h at 5 mg/kg.

Overall, the amorphous drug nanoprecipitates provided a substantial intestinal absorption enhancement for both ARV-110 and ARV-471 due to increased transport across the ABL of colloidal nanoprecipitates. This dose-proportional flux advantage through particle drifting extended approximately 14-fold above the amorphous solubility for the PROTACs (Figure 6A+B). At higher particle concentrations, further flux gains were either limited due to the very low drug solubility of ARV-110 (flux plateau) or constrained due to slower diffusion across the ABL of larger drug particles (subproportional flux advantage), as observed for ARV-471. At clinically relevant doses (>1 mg/kg), the differences in epithelial membrane permeability of ARV-110 and ARV471 are expected to play a minor role in the potential absorption enhancement of the colloidal nanoprecipitates, as amorphous solubility is exceeded, rendering solubility or dissolution rate-limiting for absorption. In contrast, at doses near or below amorphous solubility, where precipitation is minimal, and drug is rapidly dissolved or solubilized, absorption becomes permeability-limited. Accordingly, at the lowest respective doses (0.04 mg/kg for ARV-110 and 0.2 mg/kg for ARV-471), differences in P_eff_ are reflected in the substantially higher f_abs_ of the more permeable ARV-110 (0.52) compared with ARV-471 (0.17), whose absorption remains intrinsically constrained by its low P_eff_.

### 4.2 Impact of Amorphous Drug Nanoprecipitates on ARV-471 Absorption

The direct contribution of the amorphous drug nanoprecipitates to the intestinal absorption of ARV-471 was further assessed by comparing two formulations that only differed in the physical nature of the suspended particles: one containing amorphous nanocolloidal particles and the other a dispersed noncolloidal amorphous powder. Observed in vivo differences in flux could be fully attributed to the amorphous nanocolloidal particles, as the solubilized concentrations in both study groups were the same. The colloidal formulation had a 2.4-fold higher AUC_0–6h_ than the amorphous powder suspension, an effect primarily attributed to a 7-fold higher flux directly after dosing. The large size of the amorphous particles (extending into the higher μm range; Supporting Information Figure S3) limited particle drifting, and their solid state led to slower dissolution as compared to the readily dissolving amorphous drug nanoprecipitates. These findings are corroborated by a recent study by Sharma et al., which showed that a nanoparticle-forming ASD, compared to one that does not lead to the formation of nanoparticles, resulted in a more than 2-fold increase in *C*_max_ of enzalutamide.^51^ Generation of nanosized amorphous particles or precipitates can thus increase intestinal absorption well beyond the amorphous solubility threshold, opening considerable formulation opportunities to address the absorption limitations commonly observed for PROTACs.

### 4.3 Implications for PROTAC Drug Delivery

The vehicles used in this study were designed to increase absorption by mimicking ASDs, which are known to rapidly generate drug-rich colloids and amorphous drug nanoprecipitates via LLPS upon drug release and supersaturation in luminal fluids.^24^ For some compounds, however, the generation of colloidal species is a precursor to crystallization, which may ultimately reduce drug absorption.^48,60^ This was not the case for the PROTACs in this study, where amorphous, nanosized precipitates with glassy state behavior and minimal crystallization propensity were formed, even in the absence of crystallization-inhibiting polymers. The intrinsically poor crystallization tendency and the limited particle growth are characteristic for large and flexible molecules such as most PROTACs.^61,62^ Their heterobifunctional architecture confers high conformational flexibility and hinders efficient molecular packing, thereby favoring formation of the amorphous form.^63^ Due to this important PROTAC characteristic, a flux enhancement could be maintained at concentrations ∼ 15-fold (ARV-110) and ∼ 40-fold (ARV-471) beyond the amorphous solubility. This underscores the potential of particle-generating ASD-based formulations for PROTACs. Further increases for ARV-471 may even be possible with a fine-tuned selection of polymers and an optimized drug load to generate precipitates ≤ 100 nm across a broader dose range. Another promising approach to generating nanocolloidal structures that address dissolution and diffusion limitations, as for ARV-471, is the use of lipid-based formulations such as self-nanoemulsifying drug delivery systems. Studies by Rathod et al. and Saraswat et al.^64,65^ demonstrate substantial enhancement of ARV-825 dissolution and bioavailability attributable to their high solubilization capacity and efficient colloid formation. If intestinal absorption is predominantly limited by solubility, as in the case of ARV-110, this strategy may be less effective. This is because even if the formation of additional colloidal species could elevate apparent solubility, absorption remains limited since the free drug concentration in both the intestinal lumen and ABL has already reached saturation. It is worth noting that the intestinal absorption dynamics discussed are based on studies conducted under fasted state conditions. Under fed state conditions, the rate-limiting steps in absorption may differ substantially. A lower luminal pH, along with dietary lipids and elevated bile salt concentrations, can enhance apparent solubility (see FeSSIF solubility Table 5) and influence the quantity and size of colloidal species.^52,66^ Intestinal absorption may improve under fed conditions for drugs exhibiting dissolution and ABL diffusion rate limitations, as the size of the emerging particles might decrease (ARV-471). In contrast, intestinal absorption for the solubility-limited ARV-110 is expected to benefit less from fed-state conditions. Overall, ARV-110 and ARV-471 possess distinct physicochemical properties, representative of the broader chemical space of PROTACs.^67^ Therefore, the mechanistic insights from this study may be broadly applicable in the development of PROTACs, guiding rational drug delivery strategies.

## 5 Conclusions

The present work described a mechanistic in vivo evaluation of the solubility-dissolution-permeability interplay of two poorly soluble PROTACs in rats. We provided experimental evidence for the in vivo formation of luminally stable amorphous drug nanoprecipitates, demonstrated their absorption-enhancing effect, and elucidated the underlying mechanisms governing these processes. Dose escalation studies demonstrated that nanoparticle-generating formulations offered a substantial advantage by enabling particle drifting across the ABL. Through this mechanism, drug absorption could be enhanced far beyond the amorphous solubility for both PROTACs. Our data showed that these solubility limitations could be overcome as long as the mass transfer of the particles across the ABL was not saturated, or the hydrodynamic dimensions of the particles did not exceed a size where particle diffusion across the ABL became limited and ineffective. These mechanistic insights advanced the understanding of the particle drifting effect in vivo and may guide the development of enabling colloidal PROTAC formulations.

## Supplementary Material



## Notes

Contains polarized light microscopy images of the in situ emerging precipitates, particle size distributions of the studied formulations at ascending doses, detailed information on the polydispersity index and the average hydrodynamic diameter of drug precipitates, as well as a dose-proportionality analysis using the power model (PDF)

## ∇

Merck and Co Inc. 126 E Lincoln Ave, Rahway, New Jersey 07065 United States

## Notes

The animal study was approved by the local ethics committee for animal research (5.8.18-00250/2022) in Uppsala, Sweden. The animal studies were carried out in compliance with the EU regulations for animal welfare. Animals were evaluated for general welfare twice daily by the Animal Department staff.

## Notes

During the preparation of this work the authors used ChatGPT 4o in order to check spelling or grammar. After using this tool/service, the authors reviewed and edited the content as needed and take full responsibility for the content of the publication.
