# Supplementary material for: The In Vivo Effect of Amorphous Drug Nanoprecipitates on the Intestinal Absorption of the PROTACs ARV-110 (Bavdeglutamide) and ARV-471 (Vepdegestrant)
Source: Mol Pharm. 2026 May 1;23(6):3445–57. doi: 10.1021/acs.molpharmaceut.6c00207 (PMC13231420; doi:10.1021/acs.molpharmaceut.6c00207)
Supplement: Supplementary file 1 [file mp6c00207_si_001.pdf]

The *in vivo* effect of amorphous drug nanoparticles on the intestinal absorption of the PROTACs ARV-110 (bavdeglutamide) and ARV-471 (vepdegestrant)

Janis Niessen[1], Mirko Koziolek[2], Anura Indulkar[3], Thomas Borchardt[4], Markus Sjöblom[5], Mikael Hedeland[6], Hans Lennernäs[1], David Dahlgren[1,*]

[1]Department of Pharmaceutical Biosciences, Translational Drug Discovery and Development, Uppsala University, Uppsala, Sweden

[2]AbbVie Deutschland GmbH & Co. KG, Synthetic Molecules CMC R&D, Knollstrasse, Ludwigshafen am Rhein, Germany

[3]AbbVie Small Molecule CMC Development, 1N Waukegan Road, North Chicago, IL, USA

Current address: Merck and Co Inc 126 E Lincoln Ave, Rahway, NJ 07065 United States.

[4]AbbVie Product Development Science and Technology, 1N Waukegan Road, North Chicago, IL, USA

[5]Department of Medical Cell Biology, Uppsala University, Uppsala, Sweden

[6]Department of Medicinal Chemistry, Analytical Pharmaceutical Chemistry, Uppsala University, Uppsala, Sweden

*Correspondence: david.dahlgren@uu.se

1 Supplementary Material

Besides the WAXS measurements to determine the solid state of the *in situ* emerging precipitates, polarized light microscopy was applied to verify the solid state of the precipitates, both in the to-be-dosed vehicle and the vehicle diluted with ratSIF (2:1 dilution) to simulate the state after intraduodenal bolus injection.

Figure S1 shows the recorded images taken approximately 5 min after ultracentrifugation of the preparation and dispersing the resulting pellet on a glass slide. The precipitates were found to be amorphous. Weak birefringence signals observed in panel (B) are likely artifacts originating from the ratSIF bile components. Clear crystalline structures could not be observed in any of the samples.

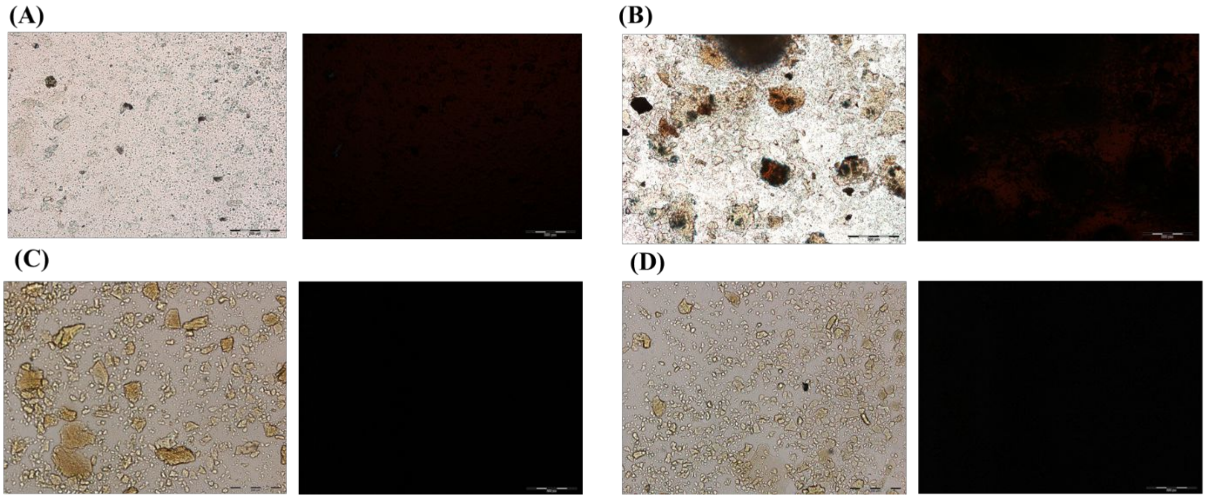

Figure S1. Images of the generated and pelleted precipitates of ARV-110 (C+D) at the highest dose of 1.0 mg/kg in the vehicle (C) and diluted in ratSIF (D) and ARV-471 (A+B) at 5.0 mg/kg in vehicle (A) and diluted in ratSIF (B) at 10x magnification. Images were taken without (left) and with a polarizer (right) to verify the solid state of the precipitates.

Figure S2 shows the number-weighted frequency distribution of the particle sizes of ARV-110 (top) and ARV-471 (bottom) at ascending doses (0.04, 0.2, 1.0 mg/kg for ARV-110 and 0.2, 1.0, 2.5, 5.0 mg/kg for ARV-471). At lower doses, the particles show monomodal narrow distributions, while at higher doses, a wider distribution was observed (ARV-471 at 5.0 mg/kg).

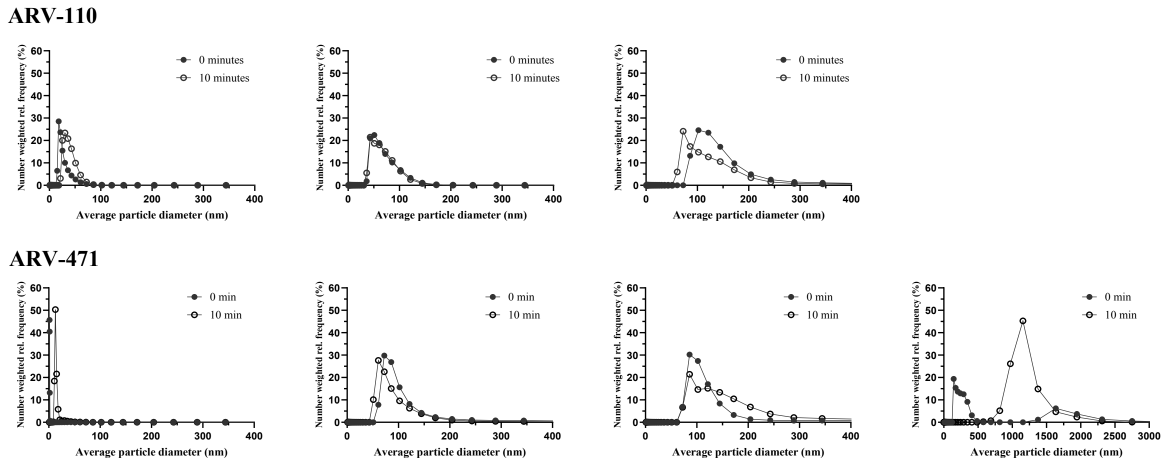

Figure S2. Particle size distributions of ARV-110 and ARV-471 at ascending doses in the vehicle prepared by solvent shifting to generate an amorphous nano-colloidal phase.

Table S 1 summarizes all measured particle sizes at all *in vivo* applied doses, including the polydispersity index (PDI) as a quantitative measure of size uniformity of the colloidal amorphous suspensions.

Table S 1. Mean ± SD hydrodynamic diameter determined by DLS for ARV-110 and ARV-471 at ascending doses at 0 min and 10 min after formulation preparation, and polydispersity index calculated from the hydrodynamic diameter as $(SD/mean)^2$.

**Dose**	**0 min**		**10 min**	
	**Hydrodynamic diameter (nm)**	**PDI**	**Hydrodynamic diameter (nm)**	**PDI**
**ARV-110**				
0.04 mg/kg	34.2 ± 6.5	0.04	44.1 ± 2.4	0.01
0.2 mg/kg	74.2 ± 15.0	0.04	64.6 ± 16.7	0.07
1.0 mg/kg	134.2 ± 15.7	0.01	116.7 ± 25.3	0.05
**ARV-471**				
0.2 mg/kg	21.9 ± 17.8	0.66	30.0 ± 15.2	0.26

1.0 mg/kg	102.0 ± 15.4	0.02	89.9 ± 19.2	0.05
2.5 mg/kg	149.2 ± 43.6	0.09	166.5 ± 40.9	0.06
5.0 mg/kg	1146 ± 748	0.42	1326 ± 451	0.12
1.0 mg/kg (powder)	1765 ± 1105	0.39	1870 ± 722	0.15

Figure S3 shows the difference between the colloidal and non-colloidal formulations of ARV-471. Particles of the non-colloidal formulation are larger, as indicated by the DLS measurements, and due to measurements exceeding the nanometer range for the non-colloidal formulation, verified by PLM measurements after dilution with ratSIF.

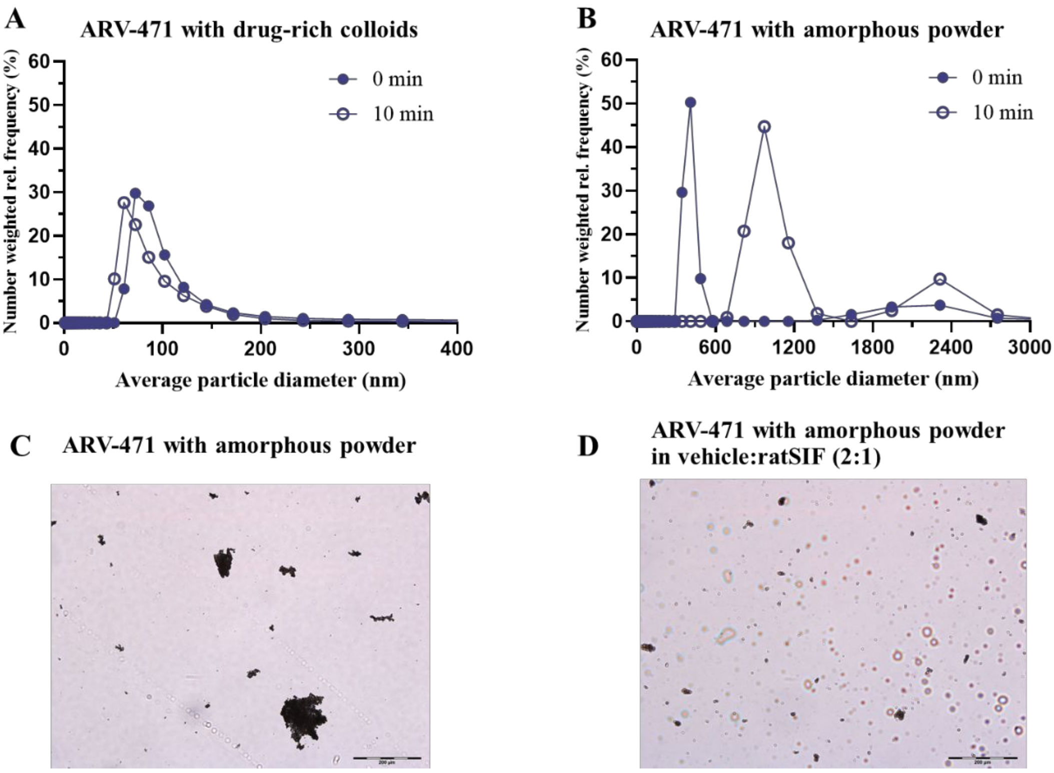

Figure S3. Particle size distributions of ARV-471 at 0.5 mg/mL (1.0 mg/kg) in the vehicle, (A) prepared by a solvent shift method to generate amorphous colloidal drug nanoprecipitates and (B) by suspending amorphous powder in the vehicle. Polarized light microscopy images at x10 magnification of the suspended amorphous powder in the vehicle (C), and in the vehicle diluted with ratSIF (D): Multiple particles exceeding far beyond the nanometer range are visible for the amorphous powder (non-colloidal) formulation.

Figure S 4 shows the results of a dose proportionality analysis using the power model to identify differences in the intestinal absorption and systemic exposure of the PROTACs depending on the administered dose.

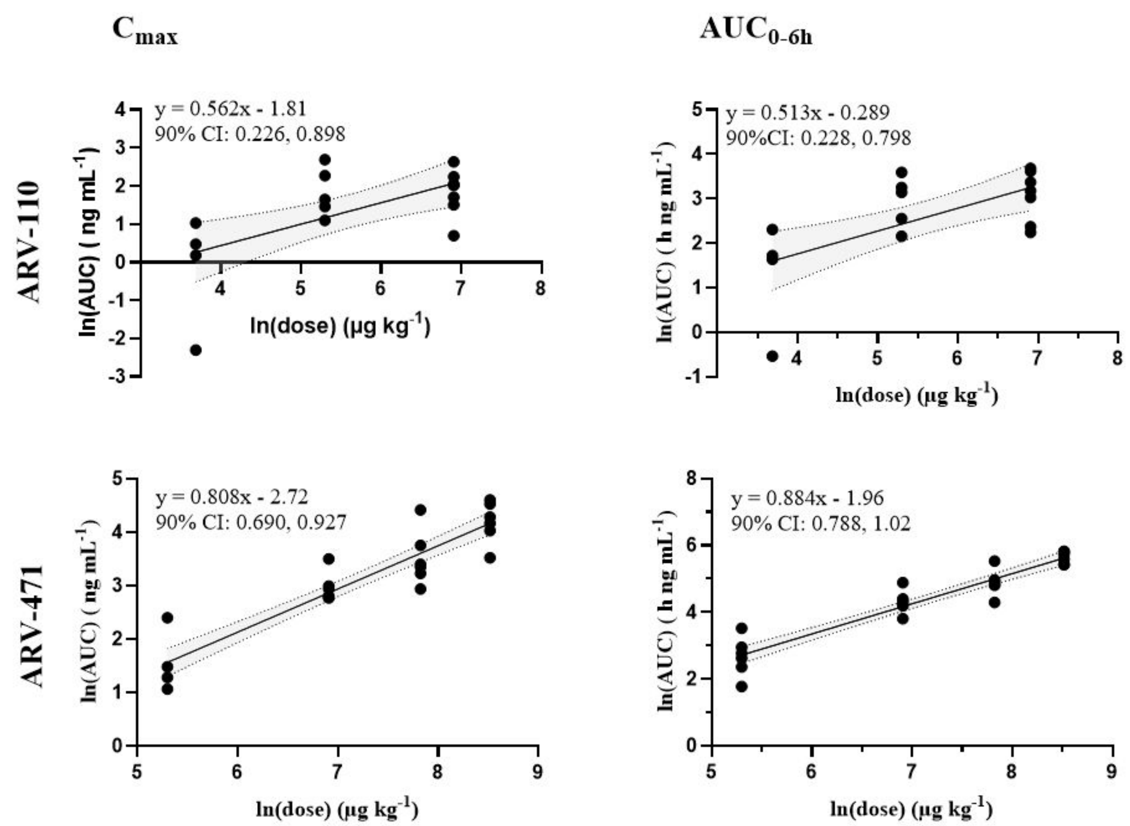

Figure S 4. Dose proportionality analysis using the power model for ARV-110 (top) and ARV-471 (bottom). The relationship ln(dose) and the natural logarithm of c_{max} (left) and $AUC_{0\text{-}6h}$ (right) is shown with individual data points, the fitted regression line, and its 90% confidence band. The equation of the fitted regression model, as well as the 90% confidence interval of the slope, is depicted for the evaluation of dose-proportional pharmacokinetics of the PROTACs.
